# Harnessing cytokines: innovative adjuvants for improved veterinary vaccine efficacy

**DOI:** 10.3389/fimmu.2025.1643855

**Published:** 2025-10-21

**Authors:** Shihong Yan, Huimin Chen, Qiao Liu, Xinyu Zhang, Qiulu Wei, Chenqi Hu, Dianfeng Chu, Hongxiu Diao, Shasha Liu, Ji-Long Chen

**Affiliations:** ^1^ Key Laboratory of Animal pathogen Infection and Immunology of Fujian Province, College of Animal Sciences, Fujian Agriculture and Forestry University, Fuzhou, China; ^2^ Joint Laboratory of Animal Pathogen Prevention and Control of Fujian-Nepal, College of Animal Sciences, Fujian Agriculture and Forestry University, Fuzhou, China; ^3^ YEBIO Bioengineering Co., Ltd, Qingdao, China

**Keywords:** cytokines, veterinary vaccine, vaccine adjuvant, immune enhancement, vaccination

## Abstract

Vaccination is one of the most effective methods for controlling animal infectious diseases, and the use of adjuvants plays crucial role in enhancing the immune efficacy of vaccines, particularly in inactivated and subunit vaccines. With the continuous advancement of research in animal immunology and immune mechanisms, our understanding of the functions of cells and cytokines in immune responses has become increasingly comprehensive, laying a solid foundation for the development of novel vaccines and adjuvants. Cytokines are a class of proteins secreted by the animal body that regulate innate and adaptive immune responses through interaction with specific receptors. To date, numerous studies have investigated the potential of using cytokines as adjuvants to enhance the efficacy of veterinary vaccines. This review focuses on cytokines as veterinary vaccine adjuvants, with special attention to the current research progress and mechanisms of cytokines such as interleukins, interferons, chemokines, and colony-stimulating factors. Additionally, examples of the application of cytokine-based adjuvants in combination with veterinary vaccines will be discussed to provide further insights and references for the development of cytokine-based veterinary adjuvants.

## 1 Introduction

Vaccination is one of the most effective strategies for controlling infectious diseases in livestock and poultry. Adjuvants, as immune enhancers, also play an indispensable role in the immunological control and prevention of animal diseases. Currently, adjuvants are widely used in the preparation of vaccines, particularly in those with weaker immunogenicity such as inactivated vaccines, synthetic peptide vaccines, subunit vaccines, and DNA vaccines (1, 2). Adjuvants can effectively reduce the number of immunizations and the amount of antigens needed, while directing the immune response towards the desired direction (3). Additionally, adjuvants help to overcome antigen competition issues in combined vaccines and improve their efficacy in immunocompromised animals (4, 5). However, the majority of veterinary vaccine adjuvants still rely on traditional adjuvants such as aluminum hydroxide and oil emulsions, which, despite their widespread use, are often associated with side effects like joint pain and muscle discomfort (6). For example, the well-known Freund’s adjuvant can cause severe adverse reactions, leading to local inflammatory lesions, pain, and discomfort. Given these limitations, there is an urgent need to develop safer and more effective new adjuvants to improve vaccine safety and immune efficacy.

Cytokines are soluble proteins produced upon stimulation by immunogens, mitogens, or other factors, and they play critical roles in signal transduction (7). By binding to specific receptors, cytokines can regulate various biological processes, including innate and adaptive immunity, hematopoiesis, cell growth, and tissue repair (8). Recent studies have demonstrated that recombinant cytokines can enhance the host resistance to disease, improve physiological functions, and maintain immune homeostasis. These findings suggest that cytokines have significant potential in enhancing vaccine efficacy and adjuvant activity.

**Figure 1:**
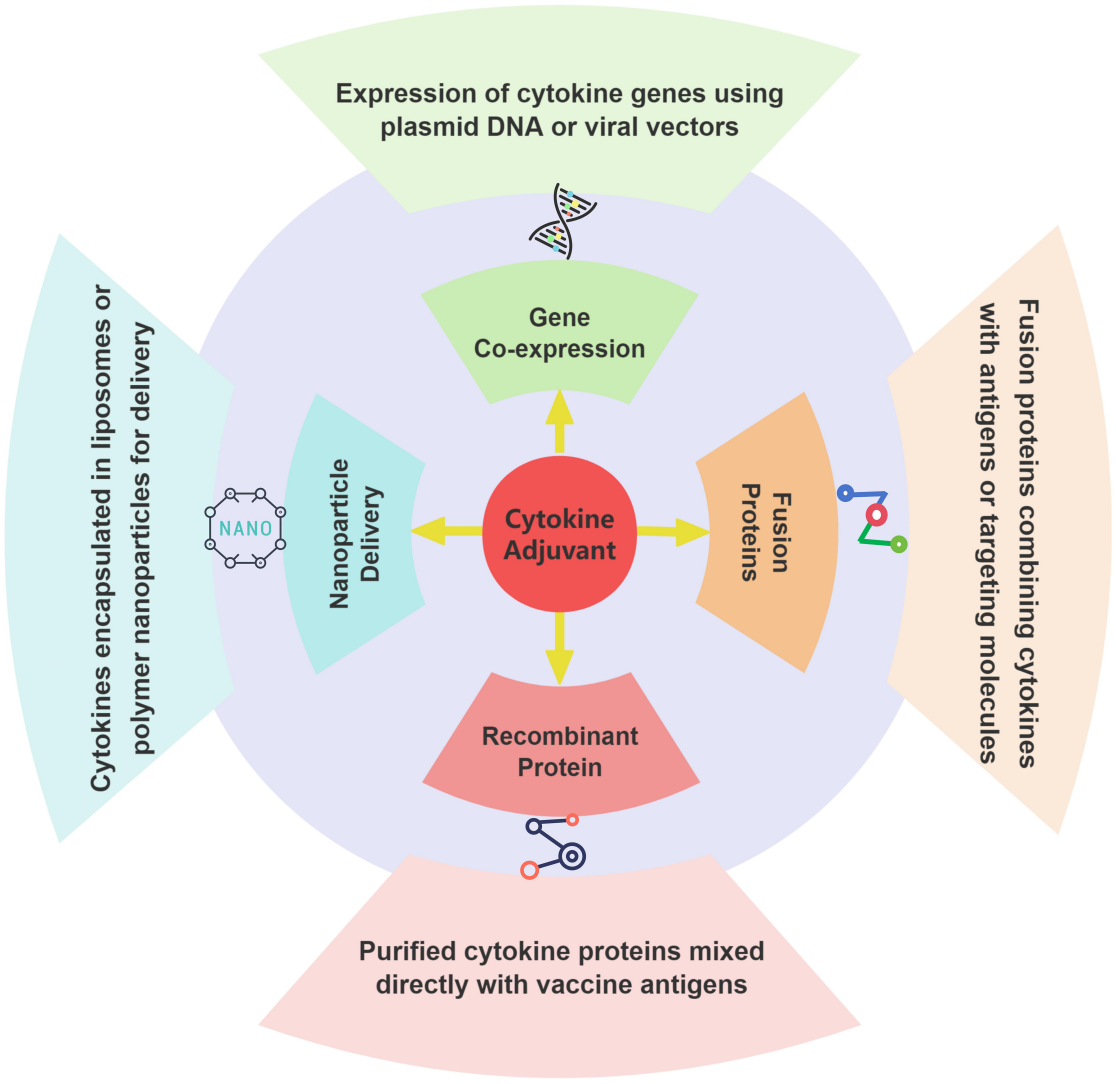
Delivery methods and carrier systems of cytokines adjuvants in veterinary vaccines.

Cytokines encompass a wide variety of molecules, including interleukins, interferons, tumor necrosis factor superfamily, colony-stimulating factors, chemokines, and growth factors. Due to their origin from the animal’s own body, cytokines are efficient, safe, and specific, with clear species specificity, which minimizes the risks of residues and adverse side effects compared to traditional adjuvants. Using cytokines as adjuvants in veterinary vaccines not only significantly enhances vaccine efficacy but also ensures the food safety of livestock and poultry products. Therefore, developing cytokine-based adjuvants is crucial for supporting the sustainable growth of the livestock industry and driving socio-economic progress. With increasing research on cytokines as vaccine adjuvants, diverse delivery methods and carrier systems have become a focus. These strategies play critical roles in cytokine stability, targeting, and immune activation. **Figure 1** provides a schematic illustration of these common approaches.

This paper discusses the current research on cytokine-based vaccine adjuvants, with a focus on the application status and adjuvant mechanisms of major cytokines such as interleukins, interferons, chemokines, and colony-stimulating factors in veterinary vaccine development. This review aims to provide theoretical references and practical guidance for the research and development of cytokine-based adjuvants.

## 2 Interleukins

Interleukins (IL) were initially described as cytokines produced by leukocytes that regulate interactions among these cells. Today, ILs refer to a family of cytokines with well-characterized molecular structures and biological functions that play critical roles in immune regulation. ILs mediate the transmission of information, activation and regulation of immune cells, as well as the activation, proliferation, and differentiation of T and B cells. They also play essential roles in inflammatory responses. **Table 1** presents the key mechanisms of cytokine-related vaccines of interleukin class, the antigens used, and the corresponding supporting literature.

**Table 1:** Comprehensive overview of cytokine-related vaccines of interleukin class: mechanisms, types, antigens, and references.

Cytokine name	Related mechanism of action	Vaccine type	Antigen	References
IL-1β	Promotes T-cell immune responses, enhances levels of IFN-γ and IL-4, and elicits higher serum antibody levels	Recombinant vaccine	Porcine Reproductive and Respiratory Syndrome Virus	(9)
	Enhances both cellular and humoral immune responses, specifically induces tissue-resident memory T cells, improves heterosubtypic immunity against influenza A viruses	Mucosal vaccine	Influenza A viruse	(10)
IL-2	Enhances the levels of FMDV-specific antibodies, increases the proliferative responses of antigen-specific spleen cells	Nanovaccine	Foot-and-mouth disease virus	(11)
	Induces higher levels of neutralizing antibodies and IL-4 expression, while reducing tissue damage upon challenge	DNA vaccine	Rabbit hemorrhagic disease virus	(12)
IL-4	Induces IgG production, promotes Th2 cell differentiation, reduces organ damage and virus shedding in vaccinated chickens	Recombinant vaccine	Newcastle disease virus	(13)
	Elevates Th1/Th2 cytokine levels, alleviates intestinal damage	DNA vaccine	Trichinella spiralis	(14)
IL-6	Increases antibody levels, enhances immune responses, reduces bacterial infection-induced damage, and significantly decreases mortality	Recombinant DNA vaccine	Vibrio harveyi	(15)
	Induces earlier and higher antibody titers, enhances vaccine immunogenicity	Inactivated RNA vaccine	Rabies virus	(16)
IL-12	Promoting Th1-type immune responses and enhancing antigen presentation, thereby synergistically boosting both humoral and cellular immunity	DNA vaccine	Newcastle disease virus	(17)
	Increases IgG antibody levels, induces a mixed IgG1/IgG2a response dominated by IgG2a, enhances IFN-γ secretion, and prolonged the survival of immunized mice	Recombinant vaccine	Toxoplasma gondi	(18)
IL-15	Supports the long-term persistence of CD8+ T cells and extends immune duration, improves levels of neutralizing antibodies and increased Th1 and Th2 responses	Inactivated vaccine	Foot-and-mouth disease virus	(19)
IL-18	Induces higher antibody titers and stronger CTL responses in guinea pigs, compensating for the limited cellular immunity	DNA vaccine	Foot-and-mouth disease virus	(20)

### 2.1 IL-1β

IL-1β directly influences the proliferation and differentiation of CD4 and CD8 T cells, particularly IL-4-producing cells, and also enhances the tissue localization and memory responses of CD8 T cells (21). The production and release of IL-1β are stimulated by pathogen-associated molecular patterns (PAMPs) or damage-associated molecular patterns (DAMPs). IL-1β as an adjuvant with a recombinant PRRSV vaccine induce a robust T-cell immune response, increase IFN-γ and IL-4 levels, and elicit higher serum antibody levels (9). This indicates that IL-1β has a dual role in enhancing both cellular and humoral immune responses. Furthermore, IL-1β serves as an effective mucosal vaccine adjuvant based on the capacity of attracting both innate and adaptive immune cells through the induction of chemokines and adhesion molecules (22), specifically inducing tissue-resident memory T cells mediated a rapid clearance of secondary IAV infections in mice, which improves heterosubtypic immunity against influenza A viruses (10).

### 2.2 IL-2

IL-2 is a cytokine that promotes the growth of bone marrow-derived T lymphocytes and was one of the first cytokines to be characterized at the molecular level (23). IL-2 enhances the growth activity of various cells, particularly the proliferation of CD4+ and CD8+ T lymphocytes (24). Additionally, IL-2 promotes the production of cytokines by natural killer (NK) cells and synergizes with IL-12 to enhance NK cell cytotoxic activity (25). In B cells, IL-2 primarily influences antibody secretion (26). There have been numerous reports on the use of recombinant IL-2 as a vaccine adjuvant (27, 28). Recombinant IL-2 encapsulated in nano-liposomes significantly enhances the levels of foot-and-mouth disease virus (FMDV)-specific antibodies and the concentrations of IFN-γ secreted from spleen cells through Th1 immune response as well as maintain longer periods of time to stimulate T and B cell proliferation and differentiation to improve antibody secretion, which successfully solves the shortcoming of a short half-life of IL-12. Additionally, it increases the proliferative responses of antigen-specific spleen cells, demonstrating its effective adjuvant properties (11). Co-expression of the IL-2 and VP60 genes in a DNA vaccine for rabbit hemorrhagic disease induced higher levels of neutralizing antibodies and IL-4 expression, while reducing tissue damage upon challenge, confirming the effectiveness of IL-2 as an adjuvant (12).

### 2.3 IL-4

IL-4 is a type I cytokine with a four-α-helix bundle structure that exhibits pleiotropic effects across multiple lineages. While IL-4 is produced by various immune cells, it is primarily secreted by activated CD4+ T cells (29). IL-4 mediates host sensitization and parasitic responses via IgE and induces IgG production, particularly IgG1 in B cells (30–32). In humans and mice, IL-4 acts as a T-cell growth factor and promotes Th2 cell differentiation. Studies have shown that recombinant Newcastle disease virus (NDV) expressing chicken IL-4 significantly reduced organ damage and virus shedding in vaccinated chickens compared to wild-type virus, indicating potential antiviral and protective adjuvant effects of IL-4 (13). As a genetic adjuvant, IL-4 co-expressed in a Trichinella spiralis DNA vaccine significantly elevated Th1/Th2 cytokine levels, alleviated intestinal damage, and demonstrated effective adjuvant functionality (14).

### 2.4 IL-6

IL-6 is a multifunctional pro-inflammatory cytokine with diverse roles in inflammation, immune responses, and hematopoiesis. IL-6 synergizes with transforming growth factor-β (TGF-β) to promote the differentiation of naïve CD4+ T cells, thereby enhancing adaptive immune responses (33). Furthermore, IL-6 promotes the production of IL-21, aids in the differentiation of T follicular helper (Tfh) cells (34) and CD8+ T cells (35), and induces B cell differentiation into plasma cells (36), thereby enhancing antibody production. The co-expression of IL-6 as the molecular adjuvant with FMDV DNA vaccine, induced a higher ratio ofIgG2a/IgG1, higher levels of expression of IFN-γin CD4+ and CD8+ T cells, IL-4 in CD4+ T cells, and *in vivo* antigen-specific cytotoxic response, which confirm both Th1 and Th2 immune response are activated (37). Both recombinant IL-6 protein and plasmids expressing the IL-6 gene have been used as adjuvants in studies on Japanese flounder (Paralichthys olivaceus). These adjuvants increased antibody levels, enhanced immune responses, reduced bacterial infection-induced damage, and significantly decreased mortality (15). Moreover, vaccination of mice with recombinant rabies virus expressing IL-6 resulted in earlier and higher antibody titers compared to the wild-type virus, demonstrating the potential adjuvant activity of IL-6 in enhancing vaccine immunogenicity (16).

### 2.5 IL-12

IL-12 is a member of the interleukin-12 (IL-12) family cytokines with an integral effect in activating cellular immune responses in mammals (38). When pathogens infect the host, IL-12 stimulates Th1 cell to release IFN-γ, promoting the Th1 cellular immune response and enhance the host’s property to clear the pathogens. For intracellular pathogens, IL-12 induces macrophages or cytotoxic T lymphocytes (CTLs) to destroy infected cells (39, 40). Macrophages exhibit strengthened activation activities based on regulation of IL-12 and upregulate the production and release of nitric oxide (NO) to further enhance the ability for antigen clearance (41). Multiple functional studies have highlighted IL-12 as a potential vaccine adjuvant with immunomodulatory properties. Co-delivery of an IL-12-expressing plasmid with an NDV F gene DNA vaccine using electroporation has been shown to significantly enhance immune responses in chickens, resulting in higher neutralizing antibody levels, increased lymphocyte proliferation, reduced viral shedding, and complete protection compared to the DNA vaccine alone (17). In addition, co-immunization with an IL-12 eukaryotic expression plasmid and a Toxoplasma gondii multi-epitope vaccine (pcROP8) enhanced the Th1 response and IFN-γ secretion, thereby providing heightened vaccine protection (18).

### 2.6 IL-15

IL-15 is a critical factor for the development, proliferation, and activation of effector NK cells and CD8+ memory T cells. It plays important roles in NK cell proliferation, cytotoxicity, cytokine production, NK cell-macrophage interactions, and the maintenance of CD4+/CD8+ memory T cell homeostasis (42). IL-15 supports the long-term persistence of CD8+ T cells and effectively extends immune duration, making it a preferred adjuvant for improving immune responses and vaccine longevity (43). The inactivated vaccine is short activities to the immune response, when bovine-derived IL-15 has been used as an adjuvant in guinea pigs immunized with an inactivated FMDV vaccine, the IL-15 adjuvanted vaccine maintained neutralizing antibody levels for up to six months in animals receiving. As well as Compared to animals immunized with the inactivated vaccine alone, those vaccinated with IL-15 adjuvants exhibited stronger Th1 and Th2 immune responses (19).

### 2.6 IL-18

IL-18, initially identified as an interferon-γ-inducing factor (44), synergizes with IL-12, mitogens, or microbial agents to promote IFN-γ production by T cells and NK cells (45–47). IL-18 also induces the expression of granulocyte-macrophage colony-stimulating factor (GM-CSF) in peripheral blood mononuclear cells (PBMCs) (44, 48–50) and stimulates IL-13 production (51). When IL-18 plasmids encapsulated in PLGA nanoparticles were used as adjuvants in combination with a foot-and-mouth disease virus DNA vaccine, they induced higher antibody titers and stronger CTL responses in guinea pigs, compensating for the limited cellular immunity often observed with inactivated FMDV vaccines (20).

## 3 Interferons

Interferons (IFNs) are a large class of cytokines that are critical in activating the immune response of the host. IFNs are categorized into three types: Type I, Type II, and Type III, all of which have the ability to activate antiviral activity by interacting with their respective receptors (52). Type I IFNs (primarily IFN-α, -β, and -ω) participate in viral clearance by inducing immune responses and provide protection against acute viral infections. Type II IFN (IFN-γ), primarily produced by activated NK cells and T cells, plays a pivotal role in both innate and adaptive immunity (53). Type III IFNs (IFN-λ1, -λ2, and -λ3) are associated with antiviral immune responses at epithelial surfaces, with their receptors being most abundantly expressed in cells of epithelial origin (54, 55). **Table 2** presents the key mechanisms of cytokine-related vaccines of interferons class, the antigens used, and the corresponding supporting literature.

**Table 2:** Comprehensive overview of cytokine-related vaccines of interferon class: mechanisms, types, antigens, and references.

Cytokine name	Related mechanism of action	Vaccine type	Antigen	References
Type I IFNs (primarily IFN-α, -β, and -ω)	Upregulates the expression of immunomodulatory cytokines such as IL-2, IL-6, IL-10, IL-18, and IFN-γ, increases the transcription of homing factors CCR9 and CCR10, induces a strong mucosal innate immune response, and enhances antibody levels	Combination vaccine	Influenza virus	(51)
	Promotes strong expression of antiviral proteins and induces specific immunity against VEEV	Recombinant vaccine	Venezuelan equine encephalitis virus	(56)
Type II IFN (IFN-γ)	Induces the expression of various immune factors, increases the production of IFN-γ and IL-4, enhances the body's immune response and antibody levels, improves the protective efficacy of the vaccine in mice	Subunit vaccine	*Hyalomma asiaticum*	(57)
	Increases survival rates, upregulates the expression of immune-related genes, and enhances antibody production	Subunit vaccine	Edwardsiella tarda (enterobacter)	(58)
	Increases neutralizing antibody titers, accelerates viral clearance, reduces clinical symptoms, and prevents highly pathogenic PRRSV infection	Inactivated vaccine	Porcine reproductive and respiratory syndrome virus	(59)
Type III IFNs (IFN-λ1, λ2, and λ3)	Regulates antiviral immunity, upregulates serum antibodies and activates the STAT signaling pathway, and enhances the immune protective effect of vaccine	DNA vaccine	Porcine reproductive and respiratory syndrome virus	(60, 61)

### 3.1 Type I IFNs

Type I IFNs possess immunomodulatory properties and can regulate the activity of other cytokines (62). They enhance the maturation and activation of dendritic cells (56, 63), promote Th1-type immune responses, and activate B cells to facilitate antibody production (64, 65). A combination of recombinant porcine IFN-α protein and inactivated influenza vaccine has been shown to significantly upregulate the expression of immunomodulatory cytokines such as IL-2, IL-6, IL-10, IL-18, and IFN-γ. This combination also significantly increases the transcription of homing factors CCR9 and CCR10, induces a strong mucosal innate immune response, and enhances antibody levels (53). In another study, immunization with a Venezuelan equine encephalitis virus (VEEV) vaccine containing an IFN-α plasmid adjuvant in mice resulted in the robust expression of antiviral proteins and induced specific immunity against VEEV (57).

### 3.2 Type II IFN

Type II IFN is primarily produced by activated Th cells and NK cells (66). It is a multifunctional homodimeric cytokine, with IFN-γ being its sole member (58, 59). The main biological function of IFN-γ is to induce the expression of various immune factors, thereby enhancing the body’s immune response. Several studies have demonstrated that IFN-γ is an effective adjuvant for veterinary vaccines. For instance, immunization of mice with a recombinant Hyalomma asiaticum rHasCPL protein subunit vaccine combined with an IFN-γ adjuvant increased the production of IFN-γ and IL-4, enhanced antibody levels, and improved the protective efficacy of the vaccine in mice (67). *In vitro* experiments have shown that the expression of porcine IFN-γ can significantly enhance the pro-inflammatory immune response in cells infected with PRRSV (68). Additionally, in Japanese flounder, the use of an IFN-γ adjuvant with an Edwardsiella tarda subunit vaccine effectively increased survival rates, upregulated the expression of immune-related genes, and enhanced antibody production (69). Furthermore, a study assessing the immunoadjuvant effects of a recombinant poIFN-γ-poGM-CSF fusion protein in an inactivated PRRSV vaccine administered to piglets found that the coadministration of poIFN-γ-linker-poGM-CSF and PRRSV KV significantly increased neutralizing antibody titers, accelerated viral clearance, reduced clinical symptoms, and prevented highly pathogenic PRRSV infection (70). This reinforces the critical role of IFN-γ and its fusion proteins in enhancing vaccine efficacy and providing protection against viral infections in veterinary medicine.

### 3.3 Type III IFN

Type III IFNs (IFN-λ1, λ2, and λ3) are structurally related to Type I IFNs and the IL-10 family (60), and are also known as IL-29, IL-28a, and IL-28b (61, 71). Their receptor, IL-28Rα, is expressed on a limited range of cells such as macrophages, peripheral blood lymphocytes, conventional dendritic cells, epithelial cells, and plasmacytoid dendritic cells (61, 72). IFN-λ primarily acts on these cell types to regulate antiviral immunity, thus possessing potential as an adjuvant to enhance immune responses. In studies where a Porcine Reproductive and Respiratory Syndrome (PRRS) DNA vaccine expressing IFNλ1 was used to immunize mice, there was an upregulation of serum antibodies and activation of the STAT signaling pathway. This suggests that IFNλ1 can enhance the immune protective effect of PRRSV DNA vaccines (73, 74).

## 4 Chemokines

Chemokines are a class of cytokines that play a significant role in inducing cell migration and motility, stimulating intracellular signaling pathways (75). They regulate lymphocyte development, activation, and effector functions and play a crucial role in immune surveillance. Many chemokines have been shown to be effective immunological adjuvants, enhancing the protective effects induced by viral, bacterial, and parasitic vaccines (76, 77). They are categorized into four major subclasses based on their conserved cysteine motifs, known as C, CC, CXC, and CX3C (78). **Table 3** presents the key mechanisms of cytokine-related vaccines of chemokines class, the antigens used, and the corresponding supporting literature.

**Table 3:** Comprehensive overview of cytokine-related vaccines of chemokines class: mechanisms, types, antigens, and references.

Cytokine name	Related mechanism of action	Vaccine type	Antigen	References
CCL4	Effectively lures CD4+, CD25+ T cells, regulates T cells	DNA vaccine	Vibrio anguillarum	(76)
CCL35.2	Upregulates the mRNA expression of key immune genes IL-1β, IL-2, IFN-γ2, and viperin in Carassius auratus gibelio, increases the levels of complement C3, lysozyme, and total superoxide dismutase, as well as enhances resistance to pathogens	DNA vaccine	Cyprinid herpesvirus 2	(77)
CCL28	eliciting a systemic mucosal immune response, significantly enhancing the host's cross-protective efficacy against heterologous viruses, producing long-lived antibodies, and improving mucosal antibody levels and local immune responses.	Chimeric virus-like particles vaccine	H3N2 subtype Influenza virus	(79, 80)
	Elicited elevated peripheral IFN-gamma and antigen-specific IgG while driving antigen-specific T-cell secretion of cytokine and antibody production,long-lived antibody responses	DNA vaccine	H1N1 subtype Influenza virus	(81)

### 4.1 CCL4

CCL4, also known as Macrophage Inflammatory Protein-1β (MIP-1β) (79), is effective chemoattractant for CD4+CD25+ T cell populations and is a phenotypic characteristic of regulatory T cells (80). CCL35.2 in crucian carp has the highest identity with mammalian CCL4. Using CCL35.2 plasmid adjuvant in combination with a DNA vaccine to immunize crucian carp can effectively upregulate the mRNA expression of key immune genes IL-1β, IL-2, IFN-γ2, and viperin in Carassius auratus gibelio. It also increases the levels of complement C3, lysozyme, and total superoxide dismutase, significantly enhancing the resistance of crucian carp to Cyprinid herpesvirus 2 (81).

### 4.2 CCL28

CCL28, also known as Mucosa-Associated Epithelial Chemokine (MEC), has unique immunoregulatory properties in various mucosal areas, attracting IgA and directing their migration to different mucosal sites (82, 83). Many chemokines are effective immune adjuvants in various model systems, enhancing protection induced by viral, bacterial, and parasitic vaccines, and regulating the direction and magnitude of induced immune responses produced by DNA, protein, subunit, or peptide vaccines (77). When used as a virus-like particle (VLP) vaccine adjuvant for influenza vaccines, CCL28 can act as an immune stimulant in a membrane-bound form, eliciting a systemic mucosal immune response and significantly enhancing the host’s cross-protective efficacy against heterologous viruses (84). Furthermore, studies have shown that the addition of a CCL28 adjuvant in H3N2 influenza vaccines can induce a significant increase in IgA levels and hemagglutination inhibition (HI) titers, enhancing long-term cross-protection against H3N2 influenza virus (85). A vaccination strategy that employs the intramuscular co-delivery of CCL27 or CCL28 has been shown to generate strong systemic and local immune responses, leading to the production of long-lived antibodies that neutralize influenza (86).

## 5 Colony-stimulating factors

Members of the CSF superfamily are involved in the generation of mammalian bone marrow cells, including monocytes, macrophages, dendritic cells, and polymorphonuclear phagocytes such as neutrophils and eosinophils. This family contains three key members: Macrophage Colony-Stimulating Factor (M-CSF or CSF-1), Granulocyte Colony-Stimulating Factor (G-CSF or CSF-3), and Granulocyte-Macrophage Colony-Stimulating Factor (GM-CSF or CSF-2). Among these, GM-CSF has been extensively studied for its potential as an adjuvant. **Table 4** presents the key mechanisms of cytokine-related vaccines of colony stimulate factor, the antigens used, and the corresponding supporting literature.

**Table 4:** Comprehensive overview of cytokine-related vaccines of colony stimulate factor: mechanisms, types, antigens, and references.

Cytokine name	Related mechanism of action	Vaccine type	Antigen	References
GM-CSF	Stimulates the proliferation and differentiation of bone marrow progenitor cells into granulocytes and macrophages, activates and maintains mature bone marrow cells, as well as plays an essential role in immune responses against a variety of pathogens	Recombinant vaccine	Mycobacterium tuberculosis, Epstein-Barr virus, Human Immunodeficiency Virus and so on	(77, 83–90)
	Elevates PCV-specific antibody levels, stimulates CD4+ and CD8+ T cell proliferation, upregulates the transcription of IL-1, IL-8, and IL-17, and also enhances immune protection	Inactivated vaccines and subunit vaccines	Porcine circovirus virus	(90)

### 5.1 GM-CSF

GM-CSF is a hematopoietic growth factor produced by various immune cells (87). It stimulates the proliferation and differentiation of bone marrow progenitor cells into granulocytes and macrophages (88), as well as activating and maintaining mature bone marrow cells (89). GM-CSF responds to immune cell survival, differentiation, and proliferation by inducing various signaling pathways, which is a key step in helping the immune system fight infections (90). GM-CSF levels significantly increase during inflammatory responses (90, 91), and multiple studies have demonstrated that T cell-derived GM-CSF plays an essential role in immune responses against a variety of pathogens, such as Mycobacterium tuberculosis, Epstein-Barr virus, and Human Immunodeficiency Virus and the rabies virus (92–96). Based on its pro-inflammatory effects induced by recruiting and activating bone marrow cells (90), recombinant pGM-CSF has been tested as a co-adjuvant with pFLIC protein in conjunction with a Porcine Circovirus (PCV) vaccine to immunize pigs. This approach significantly elevated PCV-specific antibody levels, stimulated CD4+ and CD8+ T cell proliferation, and upregulated the transcription of IL-1, IL-8, and IL-17, indicating a robust enhancement of both humoral and cellular immune responses (97). Similarly, the combination of GM-CSF and APS as a complex immunostimulant in a PRV vaccine model resulted in higher levels of PRV-specific gB and neutralizing antibodies, and concurrently increased the production of cytokines including IL-4, IL-10, IL-2, and IFN-γ (98). These findings indicate that GM-CSF-based adjuvant strategies have promising application prospects.

## 6 Other cytokines with adjuvant potential

Other cytokines reported to have potential as veterinary vaccine adjuvants include Interferon-Induced Transmembrane Proteins (IFITMs), B-cell Activating Factor (BAFF), α-Galactosylceramide (α-GalCer), Fms-like Tyrosine Kinase 3 Ligand (FLT3-L), and CD40L (**Table 5**). IFITMs: Transgenic chickens overexpressing IFITM1 can effectively resist H5N1 influenza virus infection by inhibiting viral replication within the body (99). Although most studies on chicken IFITM1 and IFITM3 functionality have been conducted *in vitro* or in chicken embryos (100), recent findings suggest that recombinant avian-derived antiviral proteins, including cIFITM1, cIFITM3, and cViperin, can serve as effective adjuvants in inactivated H9N2 subtype avian influenza vaccines (101). This highlights the potential of these proteins not only in enhancing viral resistance but also in improving the efficacy of avian influenza vaccines. BAFF: Incorporating membrane-anchored BAFF into Rabies Virus (RABV) virus-like particles (VLPs) induced higher antibody titers compared to inactivated RABV vaccines, demonstrating that BAFF is an effective membrane-anchored molecular adjuvant (102) α-GalCer: When used as an adjuvant with an inactivated H1N1 swine influenza vaccine administered intranasally, α-GalCer enhanced Th1 cytokine (IFN-γ and IL-12) secretion in the lungs, reduced the levels of immunosuppressive cytokines (IL-10 and TGF-β), and decreased lung viral loads (103). FLT3-L: Exogenous FLT3-L addition promoted the proliferation of CD141+ dendritic cells (DCs) and CD1c+ DCs in mouse blood, spleen, and bone marrow, thereby improving antigen presentation capabilities. This indicates its potential to enhance vaccine immunogenicity and promote antigen recognition, although research is currently limited and requires further exploration (104). CD40L: The co-administration of plasmid-expressed CD40L with Montanide™ GEL01 adjuvant enhanced the protective efficacy of a Bovine Herpesvirus-1 (BoHV-1) DNA vaccine. This combination increased the percentage of PBMCs and upregulated the expression of IFN-γ and IL-4 in cattle (105). The co-expression of CD40L and CD205 also receive the similar results (106).

**Table 5:** Comprehensive overview of cytokine-related vaccines of other cytokines: mechanisms, types, antigens, and references.

Cytokine name	Related mechanism of action	Vaccine type	Antigen	References
IFITM1	Resists virus infection by inhibiting viral replication within the body	Inactivated vaccine	H5N1 subtype influenza virus	(91)
	Enhances viral resistance and the efficacy of avian influenza vaccines, inhibits the replication, invasion, and spread of H9N2 AIV within the host	Inactivated vaccine	H9N2 subtype avian influenza virus	(93)
BAFF	Induces higher antibody titers, improves the speed and intensity of anti rabies antibody response, and as an effective membrane-anchored molecular adjuvant, may enhance the efficacy of currently used inactivated vaccines based on rabies.	Inactivated vaccine	Rabies virus	(94)
α-GalCer	Enhances Th1 cytokine (IFN-γ and IL-12) secretion in the lungs, reduces the levels of immunosuppressive cytokines (IL-10 and TGF-β), and decreases lung viral loads, as well as enhances the cross protective immunity of vaccines and innate and adaptive immune responses to antigens	Inactivated vaccine	H1N1 subtype swine influenza virus	(95)
FLT3-L	Improvs antigen presentation capabilities, enhances vaccine immunogenicity and promotes antigen recognition	Recombinant vaccine	No specific pathogen	(96)
CD40L	Enhances the protective efficacy of vaccines, increases the percentage of peripheral blood mononuclear cells (PBMCs) and upregulates the expression of IFN-γ and IL-4 in cattle	DNA vaccine	Bovine Herpesvirus-1	(97)

## 7 Conclusion

**Figure 2:**
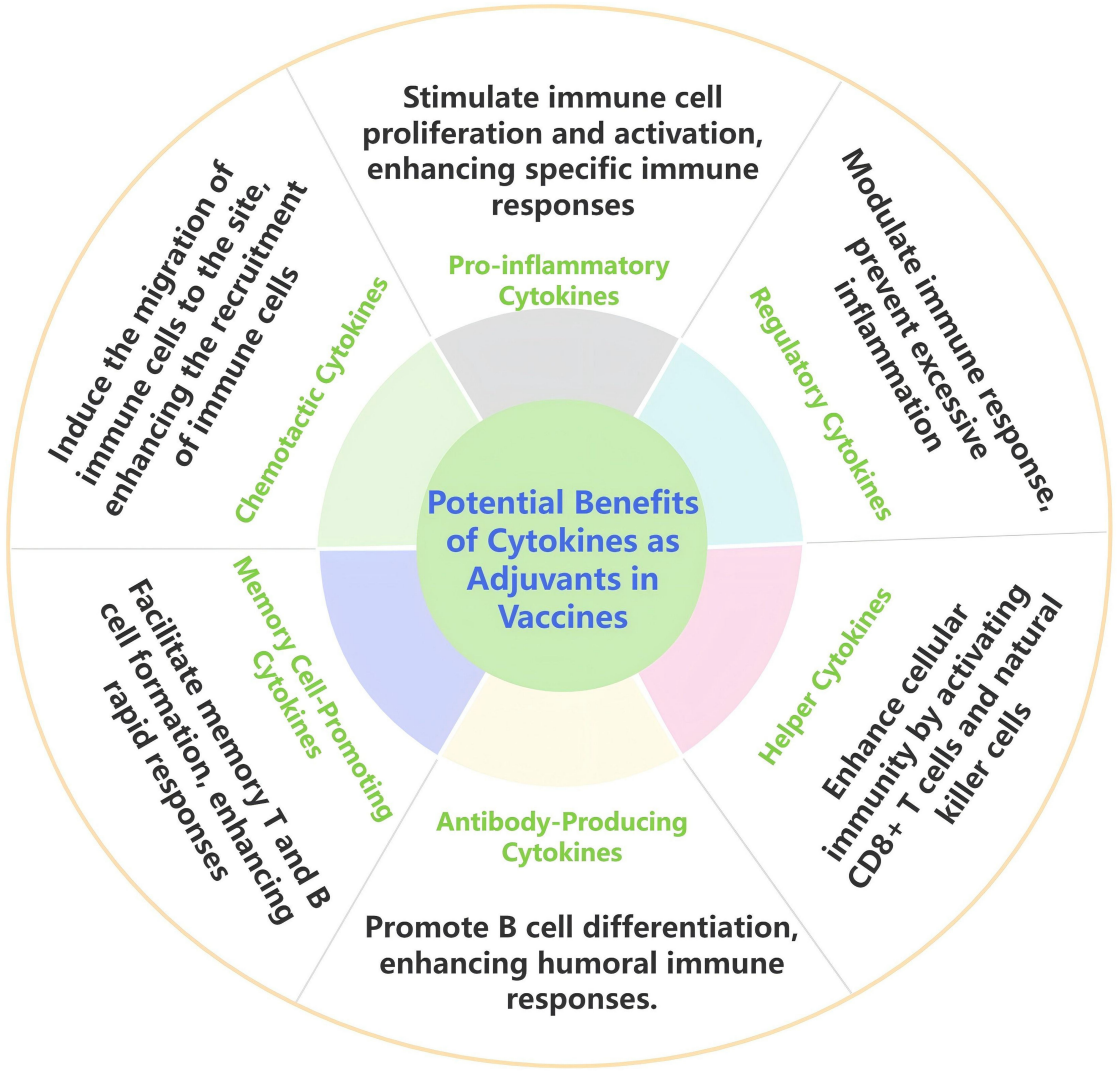
Potential benefits of cytokines as adjuvants in in veterinary vaccines.

In modern veterinary vaccine industries, vaccine adjuvants are considered crucial bridges between innate and adaptive immunity. An effective adjuvant can enhance the immunogenicity of a vaccine through various mechanisms, including promoting cytokine production, inducing antibody generation, regulating immune type switching, optimizing surface delivery to immune tissues, and improving the uptake efficiency of antigen-presenting cells. Moreover, the application of nanomaterials holds promise for further enhancing the effects of cytokine adjuvants by optimizing release characteristics and improving bioavailability, thus providing new support for immunological enhancement of vaccines (107, 108). As shown in **Figure 2**, the potential benefits of cytokines as vaccine adjuvants include different types of cytokines and their corresponding mechanisms, which play a crucial role in enhancing the immunogenicity of vaccines. This review summarizes the research and applications of different cytokines as veterinary vaccine adjuvants.

Interleukin enhances humoral and cellular immunity, induces Th1 and Th2 type responses in key effector cells, thereby prolonging the duration of immunity and improving the actual protective effect of vaccines. Interferon can upregulate the expression of immune regulatory cytokines and activate key signaling pathways such as STAT. On the one hand, it promotes high-level immune responses in mucosal areas, and on the other hand, it synergistically enhances humoral and cellular immunity at the systemic level, activating multifunctional T cell responses. In addition, interferon can stimulate strong pro-inflammatory responses while also avoiding pathological damage by regulating T cells and other mechanisms, endowing vaccines with faster onset speed, wider cross protection range, stronger pathogen clearance ability, and significantly prolonged protection duration. Chemokines can precisely regulate immune cells such as CD4+, CD25+T cells, fully activate immune molecules, induce the expression of specific antiviral proteins, enhance immune response, and increase antibody levels. GM-CSF serves as a bridge between innate and acquired immunity, activating cells to promote the quantity and function of antigen-presenting cells, and significantly increasing pathogen specific antibody levels and enhancing immune protection against pathogens. Due to their natural presence in the host organism, cytokines exhibit good biocompatibility and safe immunomodulatory effects, demonstrating significant potential as candidate adjuvants for vaccines. In recent years, numerous studies have explored the use of cytokines as vaccine adjuvants, clarifying the mechanisms by which they enhance immune responses. These studies indicate that cytokine-based adjuvants can effectively improve the immunogenicity of vaccines. Furthermore, the combination of various cytokines as adjuvants represents a promising strategy and may become a major focus for future adjuvant research and development (109, 110).

With the development of new technologies, the advancement of novel adjuvants is imperative, while their side effects must be continuously monitored to ensure vaccine safety. For low-cost veterinary vaccines, reducing the production cost of new adjuvants is particularly crucial (111). Additionally, research into the combination of nanomaterials and cytokines will further promote the application of novel cytokine-based adjuvants, supporting their feasibility in the veterinary vaccine market. However, to date, no commercialized cytokine-based veterinary vaccine adjuvants have been introduced. Given the frequent occurrence of animal diseases, future research must continuously improve veterinary vaccines and their adjuvants. This will provide new solutions to address the ever-evolving challenges posed by infectious diseases, thus enhancing animal health and the sustainable development of the livestock industry.

## References

[1] Firdaus FZ Skwarczynski M Toth I. Developments in vaccine adjuvants. Methods Mol Biol. (2022) 2412:145–78. doi: 10.1007/978-1-0716-1892-9_8, PMID: 34918245

[2] Cui Y Ho M Hu Y Shi Y. Vaccine adjuvants: current status, research and development, licensing, and future opportunities. J materials Chem B. (2024) 12:4118–37. doi: 10.1039/d3tb02861e, PMID: 38591323 PMC11180427

[3] Zhao T Cai Y Jiang Y He X Wei Y Yu Y. Vaccine adjuvants: mechanisms and platforms. Signal Transduct Target Ther. (2023) 8:283. doi: 10.1038/s41392-023-01557-7, PMID: 37468460 PMC10356842

[4] Akache B Stark FC Agbayani G Renner TM McCluskie MJ. Adjuvants: engineering protective immune responses in human and veterinary vaccines. Methods Mol Biol. (2022) 2412:179–231. doi: 10.1007/978-1-0716-1892-9_9, PMID: 34918246

[5] Schijns VEJC Strioga M Ascarateil S. Oil-based emulsion vaccine adjuvants. Curr Protoc Immunol. (2014) 106:2.18.1–2.18.7. doi: 10.1002/0471142735.im0218s106, PMID: 25081910

[6] Burakova Y Madera R McVey S Schlup JR Shi J. Adjuvants for animal vaccines. Viral Immunol. (2018) 31:11–22. doi: 10.1089/vim.2017.0049, PMID: 28618246

[7] Boyaka PN McGhee JR. Cytokines as adjuvants for the induction of mucosal immunity. Adv Drug Delivery Rev. (2001) 51:71–9. doi: 10.1016/s0169-409x(01)00170-3, PMID: 11516780

[8] Rahman T Das A Abir MH Nafiz IH Mahmud AR Sarker M. Cytokines and their role as immunotherapeutics and vaccine Adjuvants: The emerging concepts. Cytokine. (2023) 169:156268. doi: 10.1016/j.cyto.2023.156268, PMID: 37320965

[9] Lawson SR Li Y Patton JB Langenhorst RJ Sun Z Jiang Z. Interleukin-1β expression by a recombinant porcine reproductive and respiratory syndrome virus. Virus Res. (2012) 163:461–8. doi: 10.1016/j.virusres.2011.11.007, PMID: 22119401 PMC7114469

[10] Lapuente D Storcksdieck Genannt Bonsmann M Maaske A Stab V Heinecke V Watzstedt K. IL-1β as mucosal vaccine adjuvant: the specific induction of tissue-resident memory T cells improves the heterosubtypic immunity against influenza A viruses. Mucosal Immunol. (2018) 11:1265–78. doi: 10.1038/s41385-018-0017-4, PMID: 29545648

[11] Chen G Zeng S Jia H He X Fang Y Jing Z. Adjuvant effect enhancement of porcine interleukin-2 packaged into solid lipid nanoparticles. Res Veterinary Sci. (2014) 96:62–8. doi: 10.1016/j.rvsc.2013.11.017, PMID: 24374120

[12] Deng Z Geng Y Wang K Yu Z Yang PO Yang Z. Adjuvant effects of interleukin-2 co-expression with VP60 in an oral vaccine delivered by attenuated Salmonella typhimurium against rabbit hemorrhagic disease. Veterinary Microbiol. (2019) 230:49–55. doi: 10.1016/j.vetmic.2019.01.008, PMID: 30827404

[13] Marcano VC Susta L Diel DG Cardenas-Garcia S Miller PJ Afonso CL. Evaluation of chickens infected with a recombinant virulent NDV clone expressing chicken IL4. Microb Pathog. (2021) 159:105116. doi: 10.1016/j.micpath.2021.105116, PMID: 34339794

[14] Xue Y Zhang B Huang HB Li JY Pan TX Tang Y. Immunoprotective effects of invasive Lactobacillus plantarum delivered nucleic acid vaccine coexpressing Trichinella spiralis CPF1 and murine interleukin-4. Vet Parasitol. (2021) 298:109556. doi: 10.1016/j.vetpar.2021.109556, PMID: 34419708

[15] Huang P Cai J Yu D Tang J Lu Y Wu Z. An IL-6 gene in humphead snapper (Lutjanus sanguineus): Identification, expression analysis and its adjuvant effects on Vibrio harveyi OmpW DNA vaccine. Fish Shellfish Immunol. (2019) 95:546–55. doi: 10.1016/j.fsi.2019.11.013, PMID: 31704205

[16] Luo J Zhang B Wu Y Tian Q Zhao J Lyu Z. Expression of interleukin-6 by a recombinant rabies virus enhances its immunogenicity as a potential vaccine. Vaccine. (2017) 35:938–44. doi: 10.1016/j.vaccine.2016.12.069, PMID: 28089546

[17] P X Y L J L B X Q L J J. Immune effect of a Newcastle disease virus DNA vaccine with IL-12 as a molecular adjuvant delivered by electroporation. Arch Virol. (2020) 165:1959–68. doi: 10.1007/s00705-020-04669-5, PMID: 32519007 PMC7282469

[18] Foroutan M Barati M Ghaffarifar F. Enhancing immune responses by a novel multi-epitope ROP8 DNA vaccine plus interleukin-12 plasmid as a genetic adjuvant against acute Toxoplasma gondii infection in BALB/c mice. Microb Pathog. (2020) 147:104435. doi: 10.1016/j.micpath.2020.104435, PMID: 32768514

[19] Nagaraj V John L Bharatiraja S Dechamma HJ Reddy GR. Adjuvantation of inactivated Foot and Mouth Disease Virus vaccine with IL-15 expressing plasmid improves the immune response in Guinea Pigs. Biologicals. (2017) 49:23–7. doi: 10.1016/j.biologicals.2017.07.005, PMID: 28734743

[20] Yang Y Teng Z Lu Y Luo X Mu S Ru J. Enhanced immunogenicity of foot and mouth disease DNA vaccine delivered by PLGA nanoparticles combined with cytokine adjuvants. Res Veterinary Sci. (2021) 136:89–96. doi: 10.1016/j.rvsc.2021.02.010, PMID: 33592449

[21] Ben-Sasson SZ Hogg A Hu-Li J Wingfield P Chen X Crank M. IL-1 enhances expansion, effector function, tissue localization, and memory response of antigen-specific CD8 T cells. J Exp Med. (2013) 210:491–502. doi: 10.1084/jem.20122006, PMID: 23460726 PMC3600912

[22] Furuichi K Wada T Iwata Y Kokubo S Hara A Yamahana J. Interleukin-1-dependent sequential chemokine expression and inflammatory cell infiltration in ischemia-reperfusion injury. Crit Care Med. (2006) 34:2447–55. doi: 10.1097/01.CCM.0000233878.36340.10, PMID: 16849996

[23] Zhong S Zhang T Tang L Li Y. Cytokines and chemokines in HBV infection. Front Mol Biosci. (2021) 8:805625. doi: 10.3389/fmolb.2021.805625, PMID: 34926586 PMC8674621

[24] Lenardo M Chan FK-M Hornung F McFarland H Siegel R Wang J. MATURE T LYMPHOCYTE APOPTOSIS—Immune regulation in a dynamic and unpredictable antigenic environment. Annu Rev Immunol. (1999) 17:221–53. doi: 10.1146/annurev.immunol.17.1.221, PMID: 10358758

[25] McQuaid SL Loughran ST Power PA Maguire P Szczygiel A Johnson PA. Low-dose IL-2 induces CD56bright NK regulation of T cells via NKp44 and NKp46. Clin Exp Immunol. (2020) 200:228–41. doi: 10.1111/cei.13422, PMID: 31989589 PMC7232012

[26] Blackman MA Tigges MA Minie ME Koshland ME. A model system for peptide hormone action in differentiation: Interleukin 2 induces a B lymphoma to transcribe the J chain gene. Cell. (1986) 47:609–17. doi: 10.1016/0092-8674(86)90625-2, PMID: 3096574

[27] Fulcher DA Wong S. Carboxyfluorescein succinimidyl ester-based proliferative assays for assessment of T cell function in the diagnostic laboratory. Immunol Cell Biol. (1999) 77:559–64. doi: 10.1046/j.1440-1711.1999.00870.x, PMID: 10571678

[28] Li S Zhao B Wang F Wang M Xie S Wang S. Yak interferon-alpha loaded solid lipid nanoparticles for controlled release. Res Veterinary Sci. (2010) 88:148–53. doi: 10.1016/j.rvsc.2009.06.010, PMID: 19647842

[29] Iwaszko M Biały S Bogunia-Kubik K. Significance of interleukin (IL)-4 and IL-13 in inflammatory arthritis. Cells. (2021) 10:3000. doi: 10.3390/cells10113000, PMID: 34831223 PMC8616130

[30] Isakson PC Pure E Vitetta ES Krammer PH. T cell-derived B cell differentiation factor(s). Effect on the isotype switch of murine B cells. J Exp Med. (1982) 155:734–48. doi: 10.1084/jem.155.3.734, PMID: 7038025 PMC2186625

[31] Howard M Farrar J Hilfiker M Johnson B Takatsu K Hamaoka T. Identification of a T cell-derived b cell growth factor distinct from interleukin 2. J Exp Med. (1982) 155:914–23. doi: 10.1084/jem.155.3.914, PMID: 6977612 PMC2186613

[32] Maehara T Koga R Nakamura S. Immune dysregulation in immunoglobulin G4-related disease. Jpn Dent Sci Rev. (2023) 59:1–7. doi: 10.1016/j.jdsr.2022.12.002, PMID: 36654676 PMC9841035

[33] Tang Y Ma C Sun H Yang S Yu F Li X. Serum levels of seven general cytokines in acute brucellosis before and after treatment. Infect Drug Resist. (2021) 14:5501–10. doi: 10.2147/IDR.S341331, PMID: 34955644 PMC8694408

[34] Osii RS Otto TD Garside P Ndungu FM Brewer JM. The impact of malaria parasites on dendritic cell-T cell interaction. Front Immunol. (2020) 11:1597. doi: 10.3389/fimmu.2020.01597, PMID: 32793231 PMC7393936

[35] Bonin-Jacob CM Almeida-Lugo LZ Puga MAM MaChado AP Padovani CTJ Noceti MC. IL-6 and IL-10 in the serum and exfoliated cervical cells of patients infected with high-risk human papillomavirus. PloS One. (2021) 16:e0248639. doi: 10.1371/journal.pone.0248639, PMID: 33750983 PMC7984643

[36] Kishimoto T. Factors affecting B-cell growth and differentiation. Annu Rev Immunol. (1985) 3:133–57. doi: 10.1146/annurev.iy.03.040185.001025, PMID: 3933529

[37] Su B Wang J Wang X Jin H Zhao G Ding Z. The effects of IL-6 and TNF-alpha as molecular adjuvants on immune responses to FMDV and maturation of dendritic cells by DNA vaccination. Vaccine. (2008) 26:5111–22. doi: 10.1016/j.vaccine.2008.03.089, PMID: 18462845

[38] Gerber AN Abdi K Singh NJ. The subunits of IL-12, originating from two distinct cells, can functionally synergize to protect against pathogen dissemination. vivo. Cell Rep. (2021) 37:109816. doi: 10.1016/j.celrep.2021.109816, PMID: 34644571 PMC8569637

[39] S M HNC Cs M S B E A. The role of IL-12 in stimulating NK cells against Toxoplasma gondii infection: a mini-review. Parasitol Res. (2021) 120:2303–9. doi: 10.1007/s00436-021-07204-w, PMID: 34110502

[40] H W GR Y L X L. The role and potential application of IL-12 in the immune regulation of tuberculosis. Int J Mol Sci. (2025) 26:3106. doi: 10.3390/ijms26073106, PMID: 40243848 PMC11988481

[41] Hmama Z Peña-Díaz S Joseph S Av-Gay Y. Immunoevasion and immunosuppression of the macrophage by Mycobacterium tuberculosis. Immunol Rev. (2015) 264:220–32. doi: 10.1111/imr.12268, PMID: 25703562

[42] Ong CY Abdalkareem EA Khoo BY. Functional roles of cytokines in infectious disease associated colorectal carcinogenesis. Mol Biol Rep. (2022) 49:1529–35. doi: 10.1007/s11033-021-07006-4, PMID: 34981335

[43] Bhadra R Guan H Khan IA. Absence of both IL-7 and IL-15 severely impairs the development of CD8 T cell response against Toxoplasma gondii. PloS One. (2010) 5:e10842. doi: 10.1371/journal.pone.0010842, PMID: 20520779 PMC2877110

[44] Okamura H Nagata K Komatsu T Tanimoto T Nukata Y Tanabe F. A novel costimulatory factor for gamma interferon induction found in the livers of mice causes endotoxic shock. Infection Immun. (1995) 63:3966–72. doi: 10.1128/iai.63.10.3966-3972.1995, PMID: 7558306 PMC173557

[45] Micallef MJ Ohtsuki T Kohno K Tanabe F Ushio S Namba M. Interferon-γ-inducing factor enhances T helper 1 cytokine production by stimulated human T cells: synergism with interleukin-12 for interferon-γ production. Eur J Immunol. (1996) 26:1647–51. doi: 10.1002/eji.1830260736, PMID: 8766574

[46] Robinson D Shibuya K Mui A Zonin F Murphy E Sana T. IGIF does not drive Th1 development but synergizes with IL-12 for interferon-gamma production and activates IRAK and NFkappaB. Immunity. (1997) 7:571–81. doi: 10.1016/s1074-7613(00)80378-7, PMID: 9354477

[47] Okamura H Kashiwamura S Tsutsui H Yoshimoto T Nakanishi K. Regulation of interferon-gamma production by IL-12 and IL-18. Curr Opin Immunol. (1998) 10:259–64. doi: 10.1016/s0952-7915(98)80163-5, PMID: 9638361

[48] Tsutsui H Nakanishi K Matsui K Higashino K Okamura H Miyazawa Y. IFN-gamma-inducing factor up-regulates Fas ligand-mediated cytotoxic activity of murine natural killer cell clones. J Immunol. (1996) 157:3967–73. doi: 10.4049/jimmunol.157.9.3967, PMID: 8892629

[49] Dinarello CA Novick D Puren AJ Fantuzzi G Shapiro L Muhl H. Overview of interleukin-18: more than an interferon-gamma inducing factor. J Leukoc Biol. (1998) 63:658–64. doi: 10.1002/jlb.63.6.658 9620656

[50] Ushio S Namba M Okura T Hattori K Nukada Y Akita K. Cloning of the cDNA for human IFN-gamma-inducing factor, expression in Escherichia coli, and studies on the biologic activities of the protein. J Immunol. (1996) 156:4274–9. doi: 10.4049/jimmunol.156.11.4274, PMID: 8666798

[51] Hoshino T Wiltrout RH Young HA. IL-18 is a potent coinducer of IL-13 in NK and T cells: a new potential role for IL-18 in modulating the immune response. J Immunol. (1999) 162:5070–7. doi: 10.4049/jimmunol.162.9.5070, PMID: 10227975

[52] Negishi H Taniguchi T Yanai H. The interferon (IFN) class of cytokines and the IFN regulatory factor (IRF) transcription factor family. Cold Spring Harbor Perspect Biol. (2018) 10:a028423. doi: 10.1101/cshperspect.a028423, PMID: 28963109 PMC6211389

[53] Liu L Fan W Zhang H Zhang S Cui L Wang M. Interferon as a mucosal adjuvant for an influenza vaccine in pigs. Virologica Sin. (2019) 34:324–33. doi: 10.1007/s12250-019-00102-7, PMID: 30989429 PMC6599497

[54] Sommereyns C Paul S Staeheli P Michiels T. IFN-lambda (IFN-λ) is expressed in a tissue-dependent fashion and primarily acts on epithelial cells *in vivo* . PloS Pathog. (2008) 4:e1000017. doi: 10.1371/journal.ppat.1000017, PMID: 18369468 PMC2265414

[55] Zhou J Wang Y Chang Q Ma P Hu Y Cao X. Type III interferons in viral infection and antiviral immunity. Cell Physiol Biochem. (2018) 51:173–85. doi: 10.1159/000495172, PMID: 30439714

[56] Zhang X Wang S Zhu Y Zhang M Zhao Y Yan Z. Double-edged effects of interferons on the regulation of cancer-immunity cycle. Oncoimmunology. (2021) 10:1929005. doi: 10.1080/2162402X.2021.1929005, PMID: 34262796 PMC8253121

[57] O’Brien L Perkins S Williams A Eastaugh L Phelps A Wu J. Alpha interferon as an adenovirus-vectored vaccine adjuvant and antiviral in Venezuelan equine encephalitis virus infection. J Gen Virol. (2009) 90:874–82. doi: 10.1099/vir.0.006833-0, PMID: 19264673

[58] Gray PW Goeddel DV. Structure of the human immune interferon gene. Nature. (1982) 298:859–63. doi: 10.1038/298859a0, PMID: 6180322

[59] Farrar MA Schreiber RD. The molecular cell biology of interferon-gamma and its receptor. Annu Rev Immunol. (1993) 11:571–611. doi: 10.1146/annurev.iy.11.040193.003035, PMID: 8476573

[60] Pestka S Krause CD Walter MR. Interferons, interferon-like cytokines, and their receptors. Immunol Rev. (2004) 202:8–32. doi: 10.1111/j.0105-2896.2004.00204.x, PMID: 15546383

[61] Kotenko SV Gallagher G Baurin VV Lewis-Antes A Shen M Shah NK. IFN-λs mediate antiviral protection through a distinct class II cytokine receptor complex. Nat Immunol. (2003) 4:69–77. doi: 10.1038/ni875, PMID: 12483210

[62] Biron CA. Interferons alpha and beta as immune regulators–a new look. Immunity. (2001) 14:661–4. doi: 10.1016/s1074-7613(01)00154-6, PMID: 11420036

[63] Fenton SE Saleiro D Platanias LC. Type I and II interferons in the anti-tumor immune response. Cancers (Basel). (2021) 13:1037. doi: 10.3390/cancers13051037, PMID: 33801234 PMC7957896

[64] Brown IH. The epidemiology and evolution of influenza viruses in pigs. Vet Microbiol. (2000) 74:29–46. doi: 10.1016/s0378-1135(00)00164-4, PMID: 10799776

[65] R Y B Z D C. Type I interferon-mediated tumor immunity and its role in immunotherapy. Cell Mol Lifesciences : CMLS. (2022) 79:191. doi: 10.1007/s00018-022-04219-z, PMID: 35292881 PMC8924142

[66] Kerner G Rosain J Guérin A Al-Khabaz A Oleaga-Quintas C Rapaport F. Inherited human IFN-γ deficiency underlies mycobacterial disease. J Clin Invest. (2020) 130:3158–71. doi: 10.1172/JCI135460, PMID: 32163377 PMC7260033

[67] Song R Zhai X Fan X Ge T Li M Huercha. Recombinant interferon-gamma promotes immunoglobulin G and cytokine memory responses to cathepsin L-like cysteine proteinase of Hyalomma asiaticum and the efficacy of anti-tick. Vet Immunol Immunopathol. (2021) 235:110201. doi: 10.1016/j.vetimm.2021.110201, PMID: 33735822

[68] Charerntantanakul W Yamkanchoo S Kasinrerk W. Plasmids expressing porcine interferon gamma up-regulate pro-inflammatory cytokine and co-stimulatory molecule expression which are suppressed by porcine reproductive and respiratory syndrome virus. Veterinary Immunol Immunopathology. (2013) 153:107–17. doi: 10.1016/j.vetimm.2013.02.013, PMID: 23507439

[69] Wang H Guo M Tang X Xing J Sheng X Chi H. Immune adjuvant effects of interferon-gamma (IFN-gamma) of flounder (Paralichthys olivaceus) against Edwardsiella tarda. Dev Comp Immunol. (2021) 123:104159. doi: 10.1016/j.dci.2021.104159, PMID: 34081944

[70] Wang B-L Zhang S Liu Y Zhao Y-H Wang C-W Li Y. Enhancing immunogenicity and antiviral protection of inactivated porcine reproductive and respiratory syndrome virus vaccine in piglets. Am J Vet Res. (2024) 85:ajvr.24.02.0025. doi: 10.2460/ajvr.24.02.0025, PMID: 38889741

[71] Sheppard P Kindsvogel W Xu W Henderson K Schlutsmeyer S Whitmore TE. IL-28, IL-29 and their class II cytokine receptor IL-28R. Nat Immunol. (2003) 4:63–8. doi: 10.1038/ni873, PMID: 12469119

[72] Durbin RK Kotenko SV Durbin JE. Interferon induction and function at the mucosal surface. Immunol Rev. (2013) 255:25–39. doi: 10.1111/imr.12101, PMID: 23947345 PMC5972370

[73] Du L Pang F Yu Z Xu X Fan B Huang K. Assessment of the efficacy of two novel DNA vaccine formulations against highly pathogenic Porcine Reproductive and Respiratory Syndrome Virus. Sci Rep. (2017) 7:41886. doi: 10.1038/srep41886, PMID: 28157199 PMC5291100

[74] Österlund P Veckman V Sirén J Klucher KM Hiscott J Matikainen S. Gene expression and antiviral activity of alpha/beta interferons and interleukin-29 in virus-infected human myeloid dendritic cells. J Virol. (2005) 79:9608–17. doi: 10.1128/JVI.79.15.9608-9617.2005, PMID: 16014923 PMC1181545

[75] Zhang L LiWang PJ. Chemokine-receptor interactions: solving the puzzle, piece by piece. Structure. (2014) 22:1550–2. doi: 10.1016/j.str.2014.10.004, PMID: 25438668

[76] Smit JJ Lukacs NW. A closer look at chemokines and their role in asthmatic responses. Eur J Pharmacol. (2006) 533:277–88. doi: 10.1016/j.ejphar.2005.12.064, PMID: 16464446

[77] Mohan T Zhu W Wang Y Wang B-Z. Applications of chemokines as adjuvants for vaccine immunotherapy. Immunobiology. (2018) 223:477–85. doi: 10.1016/j.imbio.2017.12.001, PMID: 29246401 PMC5894355

[78] Zlotnik A Yoshie O. Chemokines: a new classification system and their role in immunity. Immunity. (2000) 12:121–7. doi: 10.1016/s1074-7613(00)80165-x, PMID: 10714678

[79] Miller M Mayo K. Chemokines from a structural perspective. Int J Mol Sci. (2017) 18:2088. doi: 10.3390/ijms18102088, PMID: 28974038 PMC5666770

[80] Xu H Xing J Tang X Sheng X Zhan W. The effects of CCL3, CCL4, CCL19 and CCL21 as molecular adjuvants on the immune response to VAA DNA vaccine in flounder (*Paralichthys olivaceus*). Dev Comp Immunol. (2020) 103:103492. doi: 10.1016/j.dci.2019.103492, PMID: 31494219

[81] Huo X Fan C Ai T Su J. The Combination of Molecular Adjuvant CCL35.2 and DNA Vaccine Significantly Enhances the Immune Protection of Carassius auratus gibelio against CyHV-2 Infection. Vaccines. (2020) 8:567. doi: 10.3390/vaccines8040567, PMID: 33019519 PMC7712643

[82] He J Thomas MA de Anda J Lee MW Van Why E Simpson P. Chemokine CCL28 is a potent therapeutic agent for oropharyngeal candidiasis. Antimicrobial Agents chemotherapy. (2020) 64:e00210-20. doi: 10.1128/AAC.00210-20, PMID: 32423961 PMC7526824

[83] Lazarus NH Kunkel EJ Johnston B Wilson E Youngman KR Butcher EC. A common mucosal chemokine (mucosae-associated epithelial chemokine/CCL28) selectively attracts IgA plasmablasts. J Immunol (BaltimoreMd : 1950). (2003) 170:3799–805. doi: 10.4049/jimmunol.170.7.3799, PMID: 12646646

[84] Mohan T Kim J Berman Z Wang S Compans RW Wang B-Z. Co-delivery of GPI-anchored CCL28 and influenza HA in chimeric virus-like particles induces cross-protective immunity against H3N2 viruses. J Controlled Release. (2016) 233:208–19. doi: 10.1016/j.jconrel.2016.05.021, PMID: 27178810 PMC4912934

[85] Mohan T Berman Z Luo Y Wang C Wang S Compans RW. Chimeric virus-like particles containing influenza HA antigen and GPI-CCL28 induce long-lasting mucosal immunity against H3N2 viruses. Sci Rep. (2017) 7:40226. doi: 10.1038/srep40226, PMID: 28067290 PMC5220311

[86] Kutzler MA Kraynyak KA Nagle SJ Parkinson RM Zharikova D Chattergoon M. Plasmids encoding the mucosal chemokines CCL27 and CCL28 are effective adjuvants in eliciting antigen-specific immunity. vivo. Gene Ther. (2010) 17:72–82. doi: 10.1038/gt.2009.112, PMID: 19847203 PMC10751736

[87] Basheer AS Abas F Othman I Naidu R. Role of inflammatory mediators, macrophages, and neutrophils in glioma maintenance and progression: mechanistic understanding and potential therapeutic applications. Cancers (Basel). (2021) 13:4226. doi: 10.3390/cancers13164226, PMID: 34439380 PMC8393628

[88] Hamilton JA. GM-CSF in inflammation. J Exp Med. (2020) 217:e20190945. doi: 10.1084/jem.20190945, PMID: 31611249 PMC7037240

[89] Lin Y-J Anzaghe M Schülke S. Update on the pathomechanism, diagnosis, and treatment options for rheumatoid arthritis. Cells. (2020) 9:880. doi: 10.3390/cells9040880, PMID: 32260219 PMC7226834

[90] M P J M S B. Granulocyte macrophage colony-stimulating factor has come of age: From a vaccine adjuvant to antiviral immunotherapy. Cytokine Growth factor Rev. (2021) 59:101–10. doi: 10.1016/j.cytogfr.2021.01.001, PMID: 33593661 PMC8064670

[91] Reynaud D. GM-CSF brings (good) memories. Blood. (2024) 143:2683–4. doi: 10.1182/blood.2024024908, PMID: 38935359 PMC11251203

[92] Santodonato L Agostino G D Nisini R Mariotti S Monque DM Spada M. Monocyte-derived dendritic cells generated after a short-term culture with IFN-α and granulocyte-macrophage colony-stimulating factor stimulate a potent epstein-barr virus-specific CD8+ T cell response. J Immunol. (2003) 170:5195–202. doi: 10.4049/jimmunol.170.10.5195, PMID: 12734367

[93] Barouch DH Santra S Tenner-Racz K Racz P Kuroda MJ Schmitz JE. Potent CD4+ T cell responses elicited by a bicistronic HIV-1 DNA vaccine expressing gp120 and GM-CSF. J Immunol. (2002) 168:562–8. doi: 10.4049/jimmunol.168.2.562, PMID: 11777947

[94] Mills KAM Westermann F Espinosa V Rosiek E Desai JV Aufiero MA. GM-CSF-mediated epithelial-immune cell cross-talk orchestrates pulmonary immunity to Aspergillus fumigatus. Sci Immunol. (2025) 10:eadr0547. doi: 10.1126/sciimmunol.adr0547, PMID: 40117345 PMC12122100

[95] Mwangi W Brown WC Splitter GA Davies CJ Howard CJ Hope JC. DNA vaccine construct incorporating intercellular trafficking and intracellular targeting motifs effectively primes and induces memory B- and T-cell responses in outbred animals. Clin Vaccineimmunology : CVI. (2007) 14:304–11. doi: 10.1128/CVI.00363-06, PMID: 17215335 PMC1828862

[96] Zhou M Wang L Zhou S Wang Z Ruan J Tang L. Recombinant rabies virus expressing dog GM-CSF is an efficacious oral rabies vaccine for dogs. Oncotarget. (2015) 6:38504–16. doi: 10.18632/oncotarget.5904, PMID: 26436700 PMC4770717

[97] Guo X Zheng Q Jiang X Wu C Zhang T Wang D. The composite biological adjuvants enhance immune response of porcine circovirus type2 vaccine. Veterinary Microbiol. (2019) 228:69–76. doi: 10.1016/j.vetmic.2018.11.015, PMID: 30593382

[98] Chen P Zhang W Cui Y Sun M Dong X Li W. Porcine GM-CSF and APS as a novel complex immunostimulant improves the immune effect of pseudorabies inactivated vaccine. Veterinary Microbiol. (2025) 304:110453. doi: 10.1016/j.vetmic.2025.110453, PMID: 40054056

[99] Rohaim MA Al-Natour MQ Abdelsabour MA El NR Madbouly YM Ahmed KA. Transgenic chicks expressing interferon-inducible transmembrane protein 1 (IFITM1) restrict highly pathogenic H5N1 influenza viruses. Int J Mol Sci. (2021) 22:8456. doi: 10.3390/ijms22168456, PMID: 34445163 PMC8395118

[100] Smith SE Gibson MS Wash RS Ferrara F Wright E Temperton N. Chicken interferon-inducible transmembrane protein 3 restricts influenza viruses and lyssaviruses in *vitro* . J Virol. (2013) 87:12957–66. doi: 10.1128/JVI.01443-13, PMID: 24067955 PMC3838109

[101] Yan S Chen Y Lin J Chen H Hu C Liu H. Recombinant avian-derived antiviral proteins cIFITM1, cIFITM3, and cViperin as effective adjuvants in inactivated H9N2 subtype avian influenza vaccines. Vet Microbiol. (2024) 298:110277. doi: 10.1016/j.vetmic.2024.110277, PMID: 39454284

[102] Plummer JR McGettigan JP. Incorporating B cell activating factor (BAFF) into the membrane of rabies virus (RABV) particles improves the speed and magnitude of vaccine-induced antibody responses. PloS Negl Trop Dis. (2019) 13:e0007800. doi: 10.1371/journal.pntd.0007800, PMID: 31725816 PMC6855436

[103] Dwivedi V Manickam C Dhakal S Binjawadagi B Ouyang K Hiremath J. Adjuvant effects of invariant NKT cell ligand potentiates the innate and adaptive immunity to an inactivated H1N1 swine influenza virus vaccine in pigs. Vet Microbiol. (2016) 186:157–63. doi: 10.1016/j.vetmic.2016.02.028, PMID: 27016770

[104] Ding Y Wilkinson A Idris A Fancke B O’Keeffe M Khalil D. FLT3-ligand treatment of humanized mice results in the generation of large numbers of CD141+ and CD1c+ dendritic cells. vivo. J Immunol. (2014) 192:1982–9. doi: 10.4049/jimmunol.1302391, PMID: 24453245

[105] Kornuta CA Langellotti CA Bidart JE Soria I Quattrocchi V Gammella M. A plasmid encoding the extracellular domain of CD40 ligand and Montanide GEL01 as adjuvants enhance the immunogenicity and the protection induced by a DNA vaccine against BoHV-1. Vaccine. (2021) 39:1007–17. doi: 10.1016/j.vaccine.2020.11.071, PMID: 33446386

[106] Njongmeta LM Bray J Davies CJ Davis WC Howard CJ Hope JC. CD205 antigen targeting combined with dendritic cell recruitment factors and antigen-linked CD40L activation primes and expands significant antigen-specific antibody and CD4(+) T cell responses following DNA vaccination of outbred animals. Vaccine. (2012) 30:1624–35. doi: 10.1016/j.vaccine.2011.12.110, PMID: 22240344

[107] Nazarizadeh A Staudacher AH Wittwer NL Turnbull T Brown MP Kempson I. Aluminium nanoparticles as efficient adjuvants compared to their microparticle counterparts: current progress and perspectives. Int J Mol Sci. (2022) 23:4707. doi: 10.3390/ijms23094707, PMID: 35563097 PMC9101817

[108] Wang K Wang H Wang X. Chiral nanomaterials as vaccine adjuvants: a new horizon in immunotherapy. Nanoscale. (2025) 17:1932–5. doi: 10.1039/d4nr03542a, PMID: 39676760

[109] Chrun T Lacôte S Urien C Jouneau L Barc C Bouguyon E. A Rift Valley fever virus Gn ectodomain-based DNA vaccine induces a partial protection not improved by APC targeting. NPJ Vaccines. (2018) 3:14. doi: 10.1038/s41541-018-0052-x, PMID: 29707242 PMC5910381

[110] Chrun T Lacôte S Urien C Richard CA Tenbusch M Aubrey N. A DNA vaccine encoding the gn ectodomain of rift valley fever virus protects mice via a humoral response decreased by DEC205 targeting. Front Immunol. (2019) 10:860. doi: 10.3389/fimmu.2019.00860, PMID: 31105695 PMC6494931

[111] Knight-Jones TJD Edmond K Gubbins S Paton DJ. Veterinary and human vaccine evaluation methods. Proc Biol Sci. (2014) 281:20132839. doi: 10.1098/rspb.2013.2839, PMID: 24741009 PMC4043076

